# Next‑generation sequencing failure rates in rare tumors: A real‑world single‑institution analysis

**DOI:** 10.3892/mi.2025.226

**Published:** 2025-03-18

**Authors:** Boris Itkin, Prashant Ajit Deshpande, Anoopa Pullanhi, Hasan Al-Sayegh, Doaa Abbas, Shoaib Al Zadjali, Ibrahim Al Haddabi

**Affiliations:** Sultan Qaboos Comprehensive Cancer Care and Research Center, University Medical City, Al Khoud, Muscat 123, Sultanate of Oman

**Keywords:** next-generation sequencing, next-generation sequencing failure, rare tumors, targeted multigene panel, whole-transcriptome sequencing, whole-exome sequencing

## Abstract

Reported next-generation sequencing (NGS) failure rates vary widely and are primarily based on studies of common tumor types. The present study aimed to estimate NGS failure rates in rare tumors and their association with preanalytical variables and sequencing methods in a single institution. Patients with sarcomas, rare carcinomas, and rare melanomas who underwent NGS between January 2022 and October 2023 were eligible for participation in the present study. NGS was performed as whole exome/transcriptome sequencing (WETS) based on hybrid capture or multigene commercial targeted panel. Clinicopathological and NGS-related data were extracted from clinical charts. Univariable logistic regression models were constructed with the outcome variable NGS failure and the following explanatory variables: Assay, sampling method, tissue type, and storage time. A total of 102 NGS reports from 86 patients with sarcomas (73.3%), rare carcinomas (16.3%), and melanomas (10.5%) were included. The median age of the patients was 40 years [interquartile range (IQR), 23-61 years]. Samples were obtained by biopsy (51%) and surgery (48%) and were collected from soft tissue (92.1%) or bone (7.9%) lesions. The median storage time was 2.5 months (IQR, 1.3-4.6 months). Targeted panel and WETS were used in 39.2 and 60.8% of reports, respectively. NGS failure due to insufficient material quantity or quality was observed in 14.7% of tests, corresponding to 4.7% of patients. Repeated testing was successful in 7 out of 8 patients. WETS was significantly associated with a higher probability of NGS failure due to the insufficient quantity or quality of material compared to targeted panel (odds ratio, 11.4; 95% confidence interval, 1.4-90.4; P=0.022). In summary, our findings suggest that the NGS failure rates in rare tumors are comparable to the rates reported in prevalent neoplasms. WETS can be associated with more frequent NGS failure than targeted panel. Retesting can often overcome the initial NGS testing failure.

## Introduction

Next-generation sequencing (NGS) of nucleic acids is increasingly being used in rare tumors for diagnosis and actionable biomarkers identification (1,2). Failed tumor NGS testing can result in the inability to provide diagnostic, prognostic, and therapeutic information, thereby increasing healthcare costs (3-5). Several factors related to NGS failure have been previously reported, including tumor type, sampling method, sample size, tumor volume, tissue type, tumor fraction, DNA yield, decalcifying procedures and the age of the paraffin blocks (3-6). The integrity and concentration of nucleic acids, along with high-quality libraries, are essential to ensuring successful NGS analysis (7,8). In published studies, NGS failure rates exhibit considerable variation, ranging from 4.1 to 22.5%, with discrepancies in the predictive factors for NGS failure reported across different investigations (3-6). Traditionally, the NGS testing process is divided into the pre-analytical, analytical, and post-analytical phases, with the pre-analytical phase being responsible for NGS failure in 90% of cases (5). However, assay-related variables can potentially modify preanalytical causes of NGS failure. For example, a larger panel size or the use of hybridization capture can increase DNA or RNA input requirements.

Most published studies in the field have predominantly included patients with prevalent tumors and data on NGS failure rates and their predictors in rare tumors are limited (3-7). In rare tumors, the frequent diagnostic challenges and absence of standardized therapeutic options may prompt physicians to utilize larger panels, often incorporating RNA sequencing, which can be particularly useful for precise sarcoma diagnosis. The present study aimed to assess the NGS failure rate in sarcomas, rare carcinomas, and melanomas, and analyze its association with pre-analytical variables and sequencing methods at a single institution in Oman. The co-primary objectives of the present study were to estimate the proportion of patients with rare tumors who experienced NGS failure and the proportion of assays that resulted in a failed report. The secondary objectives were to estimate the proportion of patients who achieved successful NGS after repeat testing and to identify predictors of NGS failure.

## Data and methods

### Study setting, design and data collection period

An observational study was conducted at the Sultan Qaboos Comprehensive Cancer Care and Research Center (SQCCCRC) in Muscat, Oman. From the institutional molecular pathology database, the list of patients treated in the Rare Tumors Program who underwent molecular profiling at SQCCCRC between January 1, 2022, and October 30, 2023 was retrieved. The study database was updated every 3-6 months. NGS tests were ordered for selected patients with rare tumors at the discretion of their physicians, based on their clinical judgment, and were performed as part of routine patient care. NGS testing was either performed at a referral laboratory (CARIS Life Sciences) or at the SQCCCRC molecular pathology laboratory.

### Eligibility

Patients of any sex aged ≥13 years, with a histopathological diagnosis of sarcomas of any grade, rare melanomas, carcinomas of unknown primary site, or rare carcinosarcomas were eligible for inclusion in the study. Ethical approval for this study was provided by the Sultan Qaboos Comprehensive Cancer Care and Research Center Institutional Review Board and Ethics Committee on November 10, 2022, (approval no. CCCRC-27-2022; Muscat, Oman). Patient consent to participate was waived due to the retrospective nature of the study.

### Procedures

For each patient included in the study, one of the following two assays was performed: i) Somatic whole exome/transcriptome sequencing (WETS) using the CARIS MI Profile™ (CARIS Life Sciences) (https://portal.caris.ai/); or ii) targeted sequencing using the 550 gene panel Oncomine™ Comprehensive Assay Plus (OCA Plus) (https://www.thermofisher.com/om/en/home/clinical/preclinical-companion-diagnostic-development/oncomine-oncology/oncomine-cancer-research-panel-workflow/oncomine-comprehensive-assay-plus.html).

Both assays were conducted on formalin-fixed paraffin embedded tissue specimens. Tumor enrichment was performed using micro-dissection (CARIS MI Profile) or macro-dissection (OCA Plus). The CARIS MI Profile assay used a hybrid pull-down panel of baits and sequencing on Illumina sequencing platforms (NextSeq™ or NovaSeq600™ https://www.illumina.com/systems/sequencing-platforms/novaseq.html). The in-house OCA Plus assay involved the preparation of libraries (DNA, 1.4MB across 501 genes; RNA, 49 driver fusions), followed by sequencing using the Ion S5plus™ platform (Thermo Fisher Scientific, Inc.) (https://www.thermofisher.com/order/catalog/product/A38195).

### Outcomes

The co-primary outcomes were the proportion of patients and the proportion of reports with failed NGS. The secondary outcome was the proportion of patients with a successful NGS after repeated testing.

### Data, variables, and statistical analysis

Clinicopathological and NGS-related data were extracted from electronic medical records. The storage time or paraffin block age was calculated as the time difference between the reporting and sampling dates. Testing was defined as failed when the reporting laboratory indicated an inability to meet the laboratory-defined quality requirements or cited an insufficient quantity or quality of DNA/RNA as stated in the NGS report. Since CARIS MI reports included results of DNA and RNA sequencing and immunohistochemistry analysis, an NGS test was defined as failed if either the DNA or RNA sequencing failed, regardless of the immunohistochemistry outcome. The study had two units of analysis: Patients and NGS reports. NGS failure rates were expressed as proportions. 95% Confidences intervals (CIs) for proportions were computed by the exact method. Continuous variables were summarized using the median and interquartile range (IQR). Univariable logistic regression models were constructed with the outcome variable NGS failure and the following explanatory variables: assay type, sampling method, source tissue, and storage time. Odds ratios (ORs) and 95% CIs were reported. All subgroup analyses were exploratory. A value of P<0.05 was considered to indicate a statistically significant difference. SAS (version 9.4; SAS Institute) and R statistical software (version 4.3; R Foundation for Statistical Computing) were used for statistical analysis.

## Results

**Figure 1:**
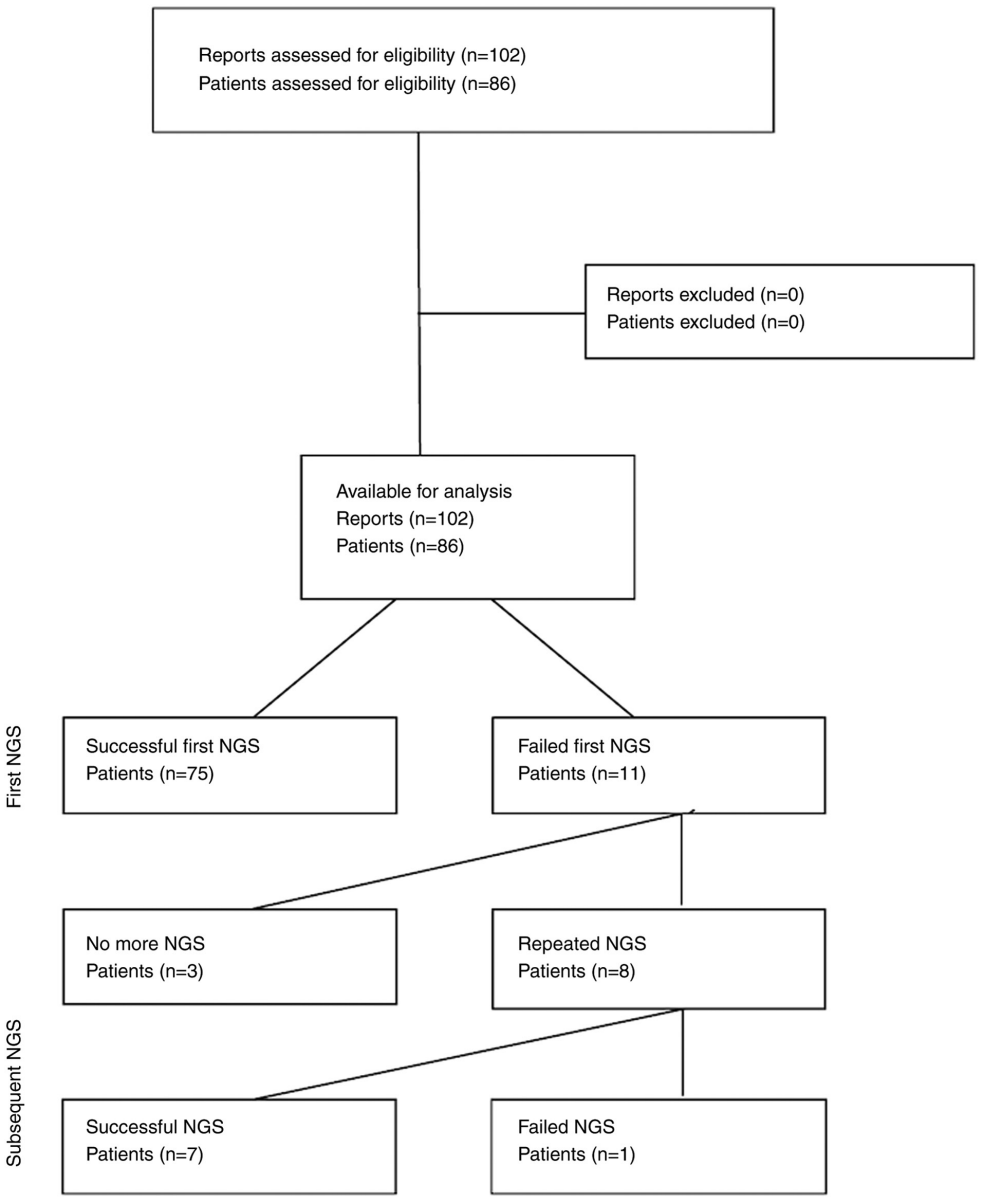
Flow diagram of the study protocol. NGS, next-generation sequencing.

A total of 102 NGS reports from 86 patients with sarcomas (73.3%), rare carcinomas (16.3%) and melanomas (10.5%) met the eligibility criteria (Fig. 1 and Table I). The median age of the patients was 40 years (IQR, 23-61 years) and 48.8% of the patients were female. A single NGS test was performed in 83.7% of the patients, while 16.3% of the patients underwent multiple NGS tests. Samples were obtained by core biopsy (51%) or surgery (48%) from soft tissue (92.1%) or bone lesions (7.9%). The median storage time was 2.5 months (IQR, 1.3-4.6). Targeted sequencing and WETS were used in 39.2 and 60.8% of reports, respectively (Table II). The sampling method, source tissue, and storage time were unavailable for 1 out of 102 reports.

**Figure 2:**
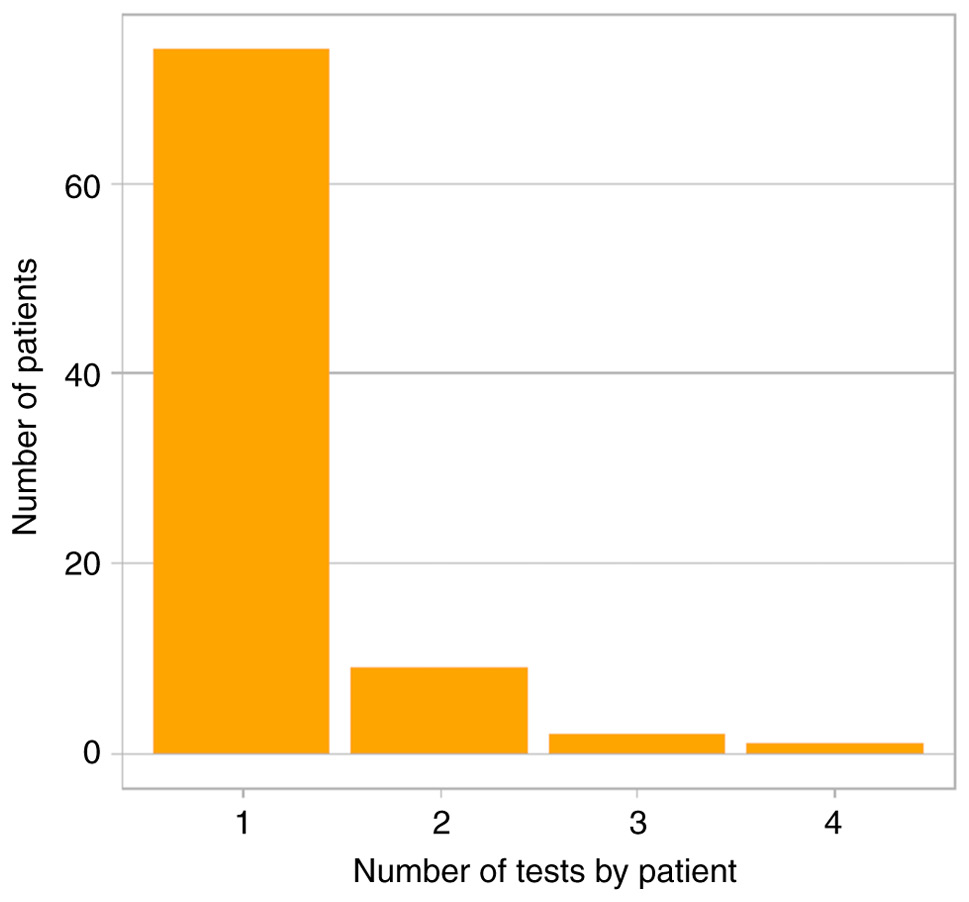
Number of next-generation sequencing tests performed on each patient.

Of the 86 patients, 87.2% had a successful initial NGS testing, while 12.8% had insufficient quantity or quality of material. Of the 11 patients with a failed first test, 8 patients underwent repeat NGS testing up to three times (Fig. 2). Repeated testing was successful in 7 out of 8 (87.5%) patients. NGS failure after any number of tests was observed in 4 out of the 86 patients (4.7%; 95% CI, 1.3-11.5%), 3 of whom had a single NGS test, and 1 patient underwent repeated NGS testing (Table SI).

Of the 102 tests, NGS failure due to insufficient material quantity or quality was observed in 14.7%; (95% CI, 8.5-33.1%), corresponding to 4/86 patients (4.7%; 95% CI, 1.3-11.5%). WETS was significantly associated with a higher probability of NGS failure due to low material quantity or quality compared with the targeted panel (OR, 11.4; 95% CI, 1.4-90.4; P=0.022; Table III). No other variable significantly predicted NGS failure. The 4 patients who did not have any successful NGS testing were diagnosed with sarcoma. The association between paraffin block age, assay type, and NGS failure is presented Fig. S1. The supporting anonymized dataset is publicly available at https://doi.org/10.5281/zenodo.14652764.

## Discussion

The present study analyzed the NGS testing failure rate in specimens from patients with rare tumors, including sarcomas, rare carcinomas and rare melanomas. It was found that NGS testing in rare tumors had a failure rate of 14.7% in the tested samples, which is comparable to that reported in more prevalent hematological and solid tumors (Table SII). To the best of our knowledge, this is the first study on NGS failure due to material quantity and quality issues specifically dedicated to the rare tumors. In our study, the NGS assay type was the only predictor of NGS failure; however, the strength of this association was imprecisely estimated, as reflected by the wide confidence interval around the odds ratio. Tumor type, sampling site and method, tissue type, and sample age were not significantly associated with the probability of NGS failure (9,10).

The small sample size of our study represents its primary limitation. Other limitations include the lack of quantification of sample volume and tumor cellularity. In a previous study on solid and hematolymphoid tumors, insufficient tumor tissue available for DNA extraction was a major factor associated with NGS failure (5). Small samples obtained from minimally invasive procedures, such as fine-needle aspiration and core needle biopsy, may yield small quantities of DNA, impacting the success rate of NGS testing. However, no significant relationship between the sampling method and the probability of NGS failure was observed in the present study. The amount of DNA needed for NGS depends on the sequencing technology, panel size, enrichment method and expected sequencing depth (9,10). While the Illumina platform successfully operates with small tissue samples containing 10-70 ng of input DNA, the Ion Torrent platform has even lower requirement for the amount of input DNA, needing only 10 ng of DNA for the Ion PGM cancer hotspot panel (5,11). In the present study, there was a significant difference between the panel size of the two assays: This was ~1.4 MB for the OCA Plus and ~30 MB for the CARIS MI Profile. Additionally, the CARIS MI Profile used the hybrid capture enrichment method whereas the OCA Plus employed the PCR. Hybrid capture enrichment has a higher DNA input requirement, particularly when compared to amplicon-based assays. It was hypothesized that the differences in the panel size and enrichment strategy contributed to the variations in failure rates observed between the two assays in this study. Regarding other potential predictors of NGS failure, the small sample size in the present study may have limited its statistical power to identify significant associations with NGS failure (12,13). Some of the potential predictors of NGS failure, such as the number of cores and specimen size, clinical setting of biopsy, the number of cores and specimen size, tumor cellularity and heterogeneity were mot analyzed in this study and should be addressed in the future research.

While both the OCA Plus and CARIS MI Profile assays screen for various types of somatic variants, including single nucleotide variants, indels, and structural variants, the OCA Plus assay cannot detect common fusions observed in certain sarcomas (e.g., Ewing sarcoma and synovial sarcoma), limiting its diagnostic utility for these subtypes. However, the majority of therapeutically relevant or actionable fusions, such as neurotrophic tyrosine receptor kinase (NTRK) 1/2/3, can be detected using the OCA Plus panel. WETS-based assays are ideal for identifying any potential gene fusions and actionable variants and accurately estimating tumor mutation burden. However, these assays require a higher quantity and quality of DNA. Thus, a customized approach is required to identify the most appropriate panel for each patient. The findings of the present study suggest that a comprehensive targeted gene panel may be preferable to WETS when the tissue sample is small, and the identification of therapeutic biomarkers is more critical than diagnosis refinement.

Several studies investigating the frequency of NGS failure and its predictors in hematologic and solid malignancies have been published to date. The substantial heterogeneity within and between study populations, sampling sites and methods, tissue processing methods (e.g., fresh tissue vs. paraffin-embedded blocks), and types of NGS assays used makes cross-study comparison or pooling challenging and may help explain the observed variation in NGS failure rates and inconsistencies among identified NGS predictors (Table SII). More homogeneous populations subjected to uniform interventions should be considered in the future studies to improve the precision of estimations and meaningfulness of conclusions.

In conclusion, the results of the present study suggest that the NGS failure rate in rare tumors is comparable to previously reported rates in more common neoplasms. The NGS failure rate was significantly higher with WETS compared to the targeted panel assay. In the majority of cases, retesting patient samples successfully overcame the initial NGS testing failure. However, the findings presented herein require validation in future studies.

## Supplementary Material

Association between paraffin block age, assay type and NGS failure. NGS, next-generation sequencing. WES, whole exome and transcriptome sequencing.

Repeated NGS testing for 11 patients with insufficient quantity or quality of material.

Selected studies reporting on NGS failure.

## Figures and Tables

**Table I:** Patient characteristics.

Characteristic	Value
Age, median (IQR)	40 (23-61)
Male sex, n (%)	44/86 (51.2%)
Diagnosis, n (%)	
Sarcoma	63/86 (73.3%)
Carcinoma	14/86 (16.3%)
Melanoma	9/86 (10.5%)
No. of tests	
Single test	72/86 (83.7%)
Repeated tests	14/86 (16.3%)
NGS failure	4/86 (4.7%)

IQR, interquartile range; NGS, next-generation sequencing.

**Table II:** Assay and sampling characteristics.

Characteristic	Value
Storage time in months, median (IQR)	2.5 (1.3-4.6)
Sampling method	
Biopsy	52/101 (51%)
Surgery	49/101 (48%)
Assay	
Targeted panel	40/102 (39.2%)
WES	62/102 (60.8%)
Tissue	
Soft tissue	93/101 (92.1%)
Bone	8/101 (7.9%)
NGS failure	15/102 (14.7%)

IQR, interquartile range; WES, whole exome/transcriptome sequencing; NGS, next-generation sequencing.

**Table III:** Univariable logistic regression of assay sample-related characteristics on the probability of NGS failure.

Explanatory variable	N	Odds ratio	Lower 95% CI	Upper 95% CI	P-value
Assay (WETS vs. targeted)	102	11.38	1.43	90.35	0.022
Sampling method (surgery vs. biopsy)	101	1.25	0.42	3.77	0.69
Tissue (bone vs. soft)	101	2.05	0.37	11.28	0.41
Storage time (1 month)^a^	101	1.04	0.98	1.11	0.18

^a^In the variable ‘Storage time’ 1 month corresponds to a odds ratio of 1.0. WETS, whole exome/transcriptome sequencing; CI, confidence interval.

## Data Availability

The data generated in the present study may be requested from the corresponding author. The supporting anonymized dataset ‘NGS Failure dataset supporting the article.xls’ is publicly available at https://doi.org/10.5281/zenodo.14652764.
