# Supplementary material for: Next‑generation sequencing failure rates in rare tumors: A real‑world single‑institution analysis
Source: Med Int (Lond). 2025 Mar 18;5(3):27. doi: 10.3892/mi.2025.226 (PMC11956127; doi:10.3892/mi.2025.226)

Figure S1. Association between paraffin block age, assay type and NGS failure. NGS, next-generation sequencing. WES, whole exome and transcriptome sequencing.

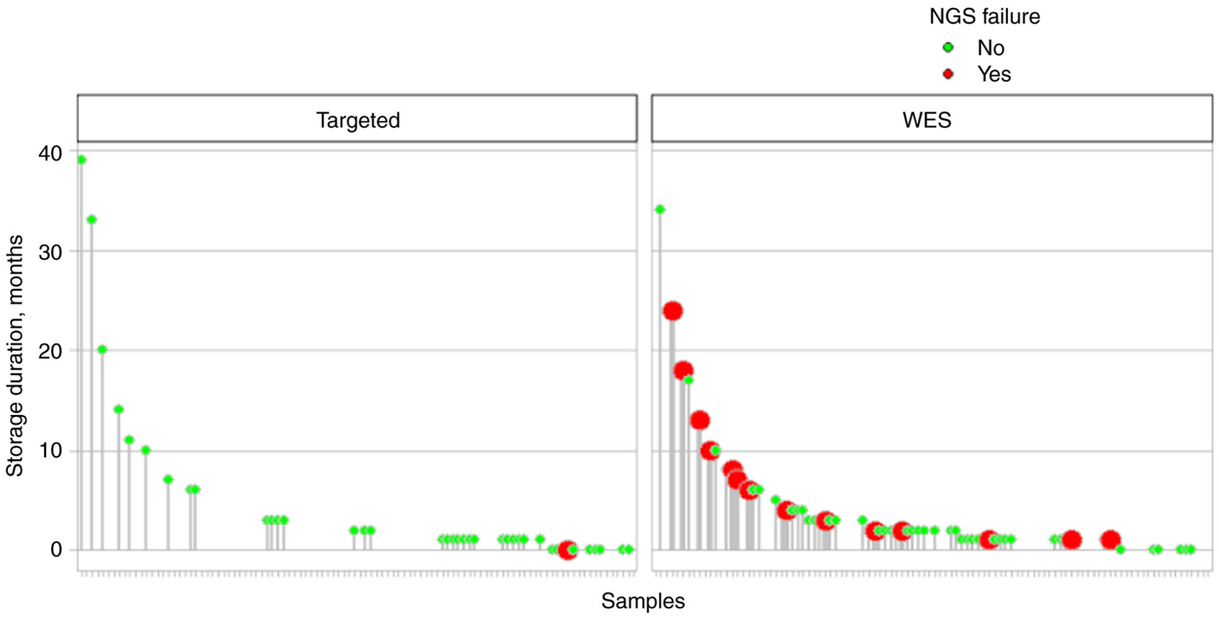

Supplement: Association between paraffin block age, assay type and NGS failure. NGS, next-generation sequencing. WES, whole exome and transcriptome sequencing. [file Supplementary_Data1.pdf]
