# Supplementary material for: Next‑generation sequencing failure rates in rare tumors: A real‑world single‑institution analysis
Source: Med Int (Lond). 2025 Mar 18;5(3):27. doi: 10.3892/mi.2025.226 (PMC11956127; doi:10.3892/mi.2025.226)
Supplement: Repeated NGS testing for 11 patients with insufficient quantity or quality of material. [file Supplementary_Data2.pdf]

Table SI. Repeated NGS testing for 11 patients with insufficient quantity or quality of material.

Patient	Initial NGS test report	NGS test report no. 2	NGS test report no. 3	NGS test report no. 4
1	Targeted (QNS)			
2	WETS (QNS)			
3	WETS (QNS)			
4	WETS (QNS)	WETS (successful)		
5	WETS (QNS)	WETS (successful)		
6	WETS (QNS)	WETS (successful)		
7	WETS (QNS)	WETS (successful)		
8	WETS (QNS)	WETS (QNS)	WETS (successful)	
9	WETS (QNS)	WETS (QNS)		
10	WETS (QNS)	WETS (QNS)	Targeted (successful)	WETS (successful)
11	WETS (QNS)	WETS (QNS)	WETS (successful)	

NGS, next-generation sequencing; WETS, whole exome/transcriptome sequencing; QNS, quantity/quality of material non-sufficient.

Table SII. Selected studies reporting on NGS failure.

Authors, year of publication	Population	No. of patients	Material and assay	NGS failure, %	Predictors of NGS failure	(Refs.)[a]
Al-Kateb *et al*, 2015	Solid and hematolymphoid neoplasms	1,528	FF and FFPE; targeted hybrid capture sequencing on Illumina platform (FFPE, bone marrow or peripheral blood)	22.5	Site of biopsy, type of biopsy, clinical setting of biopsy, specimen age, tumor heterogeneity, number of cores	(5)
Cho *et al*, 2017	Lung, GI, liver, skin, other cancers	1,564	FF and FFPE, multigene panels	4.1	Tumor type	(4)
De Silveira *et al*, 2024	Lung cancer	705	FFPE	15.1	Tumor percentage, fragment size, decalcified bone specimens, and DNA concentration	(3)
Goswami *et al*, 2016	Solid tumors: Carcinomas, melanomas, brain tumors, sarcomas, and other tumor types.	614	FFPE and cytology using the Ion Torrent platform	11	Low DNA (<10 ng) associated with small and low cellularity samples	(6)
Present study	Sarcomas, Melanomas, Carcinomas	102	FFPE	14.7	Assay type	

[a]The reference citations correspond to the reference list in the main manuscript. GI, gastrointestinal; FF, fresh-frozen; FFPE, formalin-Fixed and paraffin-embedded.
